# Movement Competency Training Delivery: At School or Online? A Pilot Study of High-School Athletes

**DOI:** 10.3390/sports8040039

**Published:** 2020-03-26

**Authors:** Simon A. Rogers, Peter Hassmén, Alexandra H. Roberts, Alison Alcock, Wendy L. Gilleard, John S. Warmenhoven

**Affiliations:** 1 School of Health & Human Sciences, Southern Cross University, Lismore 2480, NSW, Australia; peter.hassmen@scu.edu.au (P.H.); alison.alcock@equestrian.org.au (A.A.); Wendy.Gilleard@scu.edu.au (W.L.G.); 2 Applied Technology and Innovation, Australian Institute of Sport, Bruce 2617, ACT, Australia; alexhroberts17@gmail.com (A.H.R.); John.Warmenhoven@ausport.gov.au (J.S.W.); 3 Sport and Exercise Science, La Trobe University, Melbourne 3086, VIC, Australia; 4 Exercise & Sport Science, University of Sydney, Sydney 2006, NSW, Australia

**Keywords:** strength and conditioning, youth sport, neuromuscular training, athlete perceptions, training compliance, self-directed training, interviews

## Abstract

Movement competency (MC) development of high-school athletes can prepare them for the requirements of physical preparation training and the demands of sport. The aim of this study was to explore the physical effects of and athlete compliance to coach-led versus self-directed training approaches in this population. Thirty-nine high-school athletes (19 male, 14.5 ± 0.3 years old; 20 female, 14.6 ± 0.3 years) were allocated into two groups for a physical preparation program to improve MC. Groups were prescribed either (i) one face-to-face and one online (F2F, *n* = 18), or (ii) two online (OL, *n* = 21) sessions per week for 16-weeks. Before and after the intervention, the Athlete Introductory Movement Screen (AIMS) was used to assess MC alongside common physical capacity measures (triple-hop, star-excursion balance, medicine ball throw, 40 m sprint and countermovement jump). Dropout left 22 participants with pre-post physical scores. Compliance with online training was low and F2F session attendance moderate. Semi-structured interviews were conducted to assess participant perceptions following the intervention. Assessing individual responses, the F2F group had a higher proportion of positive responders to AIMS scores, yet capacity measures were inconclusive across groups. Face-to-face coaching when acquiring MCs as part of physical preparation, may provide greater positive perceptions towards training compared to self-directed online prescriptions, and thereby greater compliance.

## 1. Introduction

The importance of athletic movement competency (MC) for safe, effective and long-term physical development has been highlighted by both reviews and consensus papers [1,2]. Establishing appropriate training practices early in the youth sporting pathway is critical to maximising physical readiness outcomes, for both lifelong participation and high-performance sporting pathways [3]. Appropriate and relevant training includes the teaching and practising of fundamental movement skills to facilitate well-rounded athleticism, in addition to enhanced sport-specific skills [4]. Overlooking a strong base of MC for aspiring young athletes to physically succeed in sport, appears to play a primary role in injury risk [3]. The progression of appropriate training activities is therefore critical to athlete development and should begin with the MCs underpinning basic human movements [5].

A range of training approaches, including strength, balance, speed and agility tasks, can lower injury risk in sport [6,7,8,9]. Integrative neuromuscular training, as described by Myer et al. [3], is a mode of training defined as supplemental training, which incorporates general (i.e., building fundamental MC) and specific (i.e., exercises targeted to motor control deficits) strength and conditioning activities. Recent literature has also suggested many youth athletes selected to pre-elite squads may arrive into talent development environments below expected levels of MC to capitalise on their next stage of performance requirements [10,11,12]. Youth athletes can, therefore, benefit from earlier exposure to athletic MC skills as part of integrative neuromuscular training, to enable safe and deliberate improvements in the physical capacity to cope with the demands of sport. However, many developing high-school athletes and their often volunteer or novice coaches, are not provided with the education or full complement of resources to enable a holistic, best-practice approach to physical preparation for long term athlete development.

Typically, youth athlete training programs described in the literature have occurred in controlled and ‘mandated’ environments such as a scheduled class time (e.g., Pichardo et al. [13]) to ensure maximum attendance. Research exploring self-selected physical preparation training sessions outside of class time or sports specific practices are less documented in the current literature. Within numerous athletic intervention studies targeting adolescents, athletes’ perceptions or opinions of the training structure and its practicality within their individual sporting landscape are often overlooked. On the other hand, when supplementary training for sport is voluntary, during what would otherwise be free-time, barriers to participation are commonly assumed [14], rather than reported alongside training adherence data. Previously, Klusemann, Pyne, Fay and Drinkwater [15] performed a study investigating whether lack of access to appropriate resistance training resources (including qualified coaches) could be partly overcome via online strength and conditioning programming. Using the Functional Movement Screen™ (FMS™) [16] as their pre-post assessment tool, Klusemann and colleagues found improvements in FMS™ sum-scores that were substantially greater in the supervised athletes, over both the online video-based training and control groups. Nonetheless, the appropriateness of the FMS™ to assess youth athletes’ holistic MC and its relationship with other performance tests has been inconclusive [17]. Additionally, the environment and context surrounding Klusemann et al.’s training delivery was a single-sport talent environment (State-level squad). In this scenario, the participating basketball players (45% males: 13–15 years; 55% females: 14–16 years) were likely informed of the linkage between the prescribed training and how it might improve their basketball. Klusemann et al. [15] suggested that face-to-face coaching can provide additional verbal and kinaesthetic feedback to aid in athlete learning, and this may have a greater impact in modifying movement patterns over self-directed training methods. This work appears to be the only study to-date with comparable online and face-to-face athletic preparation training with a deliberate measure of MC.

One limitation of the studies that report positive outcomes following various forms of physical training (see Lesinksi et al. [18] for a recent review) is the majority do not assess and report the quality of movement patterns trained, or the process outcomes of training. In a recent systematic review [19], we found only eight studies involving youth athletes and reporting a process-orientated assessment of MC in training interventions of varying lengths. For example, Wright et al. [20] utilised the FMS™ in early high-school athletes to assess MC following a four-week intervention (one lunchtime session per week). The training group, however, showed only small differences in FMS™ scores (0.2 ± 1.2 points) compared to a control group, despite their high dosage of training time. While in a study of gymnasts (6–12 years of age) who performed structured integrative neuromuscular training [21], improvements in two tasks selected from the FMS™ (deep over-head squat, in-line lunge) were noted in comparison to a control group after an eight-week intervention. These examples highlight the uncertainty surrounding the time course of MC gains, and the use of differing tools to detect measurable changes in youth athletes MC. Nonetheless, including some assessment of movement *quality* is highly relevant to understand technical skill adaptation in studies assessing youth physical capacity development (e.g., of strength or power measures), particularly in cohorts without prior resistance training experience. In order to address this point, a fit-for-purpose MC screening tool was recently created. The Athlete Introductory Movement Screen (AIMS), [22] based on the work of McKeown and colleagues [23] who previously produced a more in-depth and targeted senior athlete screen the entitled the Athletic Ability Assessment. The AIMS can aid in assessing junior athletes’ readiness to engage in formal strength and conditioning activities.

In the present study, we aim to pilot and compare two delivery methods of general physical preparation sessions for high-school athletes, utilising an assessment of MC (process-orientated outcomes) alongside common physical capacity tests (product-orientated outcomes). The secondary aim is to explore the perceptions of athletes regarding the delivery of face-to-face (F2F) and/or online (OL) programs outside of class time. Specifically, we sought to explore the current program’s barriers to participation and the athlete’ experience with the non-sport specific training modalities (i.e., multimodal strength and conditioning) aiming to improve MC and physical performance.

## 2. Materials and Methods

This investigation assigned participants to one of two physical preparation intervention groups in order to explore the effectiveness of the delivery methods. Two groups were prescribed matched, twice-weekly training sessions (each 25–30 min) via two delivery variations for 16 weeks. At the start of each week, group one (F2F) was offered one session face-to-face in the school’s lunch break and a second session from an online, web-based platform (details below). The second group (OL) were provided the same two sessions to complete but solely instructed only from the online resource and to be completed in location and time of their choosing. We used a sequentially designed mixed-methods approach to augment the physical competency and capacity results of the intervention, with the qualitative perceptions of the participations [24]. By combining qualitative interview data with quantitative testing results, we were able to explore the subjective context of compliance rates and provide evidence-based recommendations for future research in this area.

### 2.1. Participants

**Figure 1:**
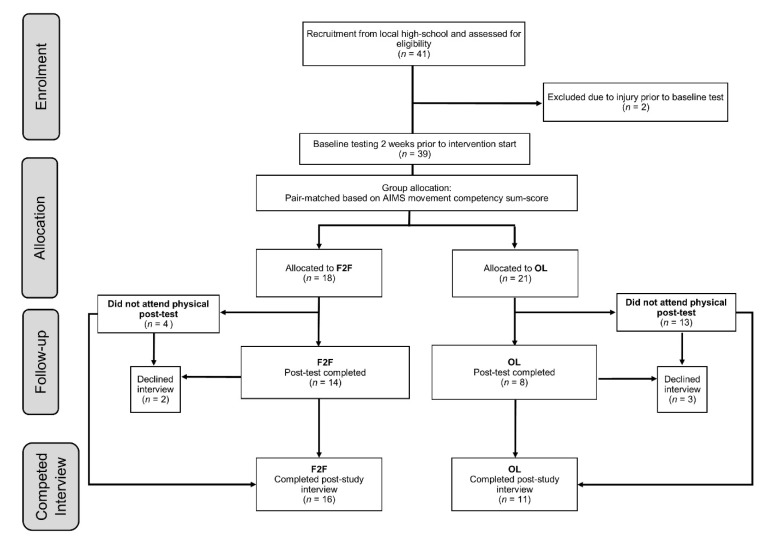
Study participant flow diagram. AIMS = Athlete Introductory Movement Screen; F2F = intervention with one session per week face-to-face and one online; OL = Intervention with online sessions only.

Thirty-nine athletes (19 males: age = 14.5 ± 0.3 years, standing height = 171.8 ± 9.7 cm, body mass 61.8 ± 12.3 kg, maturity off-set = 0.8 ± 0.7 years; 20 females: age = 14.6 ± 0.3 years, standing height =166.2 ± 6.1 cm, mass 57.4 ± 8.5 kg, maturity off-set 2.3 ± 0.4 years) from a local high-school initially volunteered for this study. Athletes were eligible for inclusion if they were currently participating in competitive sport (training and competition requiring two or more days per week) and reported no new injuries or ongoing rehabilitation in the two weeks prior to the study pre-test. The study was conducted in accordance with the Declaration of Helsinki, and ethical approval was granted by the Southern Cross University Human Research Ethics Committee (approval code ECN-16-296). Participants received detailed information and returned consent forms signed by both the athlete and their parent/guardian. As intervention training sessions were prescribed outside of their normal physical education and sport-specific training environments, all training involvement was voluntary. Because of large participant drop out, the final sample available for a pre-post analysis was 22 (see Figure 1). Subsequently, from those who initially enrolled, a total of 27 agreed to take part in post-training interviews.

### 2.2. Procedures

The following tests were performed two weeks pre- and two weeks post-intervention. All tests were performed indoors using the same equipment and protocols and conducted by the same researchers. Three days prior to pre-testing, participants performed a brief familiarisation session. Testing order was consistent across sessions in the order described below.

#### 2.2.1. Anthropometric Measures and Warm-Up

Standing and seated heights, body mass and leg length [25] were recorded by an ISAK (International Society for the Advancement of Kinanthropometry) accredited researcher. Leg length was used to normalise reach distance in the star-excursion balance test (see below). Heights, leg length and mass were used to calculate an estimated maturity off-set (age of peak height velocity) using sex-specific equations as per [26]—see Appendix A. Following anthropometrics, participants completed a standardised warm-up prior to physical testing.

#### 2.2.2. Movement Competency Tool

To assess MC in common resistance training activities, the Athlete Introductory Movement Screen (AIMS) was employed [22]. This tool helps to assess junior athletes’ readiness to engage in formal strength and conditioning activities and includes an overhead squat, push-up, lunge, and a prone brace with alternating single-hand touches to the opposite shoulder. To standardise delivery and instruction across testing sessions, all participants viewed the same pre-recorded video demonstrations with desired techniques described via key points from the scoring criteria. Following further self-guided familiarisation (1–2 min per movement), athletes performed two sets of four repetitions of each movement (as per [22]). Movements were filmed (S9, Samsung Electronics, Suwon, South Korea), recording 60 frames per second, with the first set of movements performed in the frontal plane and the second set in the sagittal plane. The primary researcher (an accredited sports scientist with 5+ years of experience in youth movement screening) assessed all athletes retrospectively from the video footage. Briefly, scores of the four movements are based on four criteria per movement worth three points each, for a total of 12 points per movement, thus 48 possible points for the overall sum (for detailed scoring criteria, see [22]).

#### 2.2.3. Star-Excursion Balance Test (SEBT)

To assess dynamic balance, the modified SEBT was performed as described by Plisky, Rauh and Kaminski [25]. Athletes were assessed without shoes and reach directions were recorded anteriorly, posteromedially and posterolaterally to the nearest whole centimetre. For each leg, the greatest distance from the three reach directions was used to produce a limb composite score (the sum of best reach in each direction, divided by three-times the measured leg length as per [25]) which was used for analysis.

#### 2.2.4. Countermovement Jump

Following three familiarisation attempts, each participant performed five maximal countermovement jumps on a 600 × 900 mm force platform (9287C, Kistler Instruments Ltd., Winterthur, Switzerland) sampling at 1200 Hz. Raw data were captured using BioWare (BioWare, 5.3.0.7, Kistler, Winterthur, Switzerland). Take-off was defined as the time when vertical ground reaction force (vGRF) was <10 N and landing when vGRF was >10 N. The raw force-time data were extracted and used to compute estimated jump height [27], with the highest jump from the session used for analysis. Participants were instructed to take-off from a self-selected stance and jump for maximum height, with hands placed on hips and legs remaining extended during flight phase [28]. Each attempt was separated by 30 s rest. If jump technique criteria were not met, the trial was repeated until the athlete completed a total of five successful jumps.

#### 2.2.5. Triple Hop

Unilateral horizontal leg power and the ability to stabilise was assessed via a triple hop for distance as outlined previously by Munro and Herrington [29]. Briefly, the triple hop involved participants performing three consecutive and maximal hops along a solid floor marked line, adjacent to a tape measure. The test began with the toes of the testing leg on the marked start line and the distance hopped measured to the rear of the same foot upon the third landing. The total distance hopped on each leg was captured to the nearest cm using a field tape. There were no restrictions on athletes regarding the use of arm swing. For a trial to be valid for recording, athletes were required to ‘stick’ the final single-leg landing for 2 s.

#### 2.2.6. Seated Medicine Ball Throw

A seated medicine ball throw was used to assess upper-body capacity as described previously [30]. Athletes sat with their legs extended with hips and back flush against a wall and were instructed to push (chest throw) a 3 kg medicine ball as far as possible. Following two familiarisation attempts, three maximal efforts were performed, with the best distance measured to the nearest centimetre using a field tape.

#### 2.2.7. 40 m Sprint Test

Participants completed 40-m sprints on a synthetic indoor track with times recorded using infrared timing gates (Fusion Sport, Coopers Plains, Brisbane, QLD, Australia). Participants completed three warm-up runs, one at each of 50%, 80% and 90% of their perceived maximal effort, with a walk back recovery, before three maximal effort attempts were recorded separated by 4–5 min passive recovery. To eliminate the influence of reaction time, athletes self-started each trial. The fastest time was used for analysis.

### 2.3. Training Program

Prior to starting the training program, an information session was presented to all intervention participants, outlining how improving MCs as part of their athlete development could enhance sporting performance and help reduce injury risk factors. The research team made individualised recommendations regarding suggested days to perform the self-directed training sessions. This was based on the athlete’s school and sporting commitments which were self-identified by athletes pre-study (self-report Summary in Table 1). Athletes were advised not to attempt or to continue with an exercise or training session if experiencing any pain. Allocation into the two training groups described above was conducted by pair-matching from the AIMS sum-scores after pre-testing, as reported previously [20]. Groups were prescribed two matched sessions to perform each week (20–30 min per session) based on common prescription levels in this age group (see [19]). For the F2F group, the first of the two weekly sessions was undertaken in the school lunch break under the guidance of a researcher (S.A.R.). Because of the in situ nature of this work and timetabling restrictions in the school, the lead researcher was only able to deliver one face-to-face session per week. The second session for the F2F group was set to be performed at home by accessing the online program following reminder emails. Athletes in the OL group had access to both the weekly sessions online. All online sessions were designed to be self-directed, housed on a custom-built online-learning website (Open Learning, Sydney, Australia). The web platform contained exercise prescription lists and video demonstrations of each exercise, accompanied by the key points for each task. The training was designed to follow an integrative neuromuscular approach, with a focus on achieving technical competence in foundational aspects of physical preparation in line with previous recommendations for youth athletes [2]. Session content was designed by Australian Strength and Conditioning Association accredited practitioners. Each session included some elements of squatting, push and/or pull variants, trunk muscular endurance, unilateral jumps with a focus on stabilisation (entry-level plyometric drills) and fundamental running technique drills (e.g., skipping variations). These were arranged into mixed-session programs to ensure athletes were exposed to all elements across the duration of the intervention period (see Appendix B for an example training session). During sessions with the F2F group, S.A.R. ensured each exercise was completed safely and with technical competency. In the majority of sessions, participants only demonstrated competency levels appropriate for bodyweight training. However, in selected tasks, such as squats, small dumbbells were used as progressions where appropriate. During the training period, participants were instructed to continue with their regular sports training and competition and to report any adverse events or injuries that occurred as a result of sports participation and/or the training intervention.

### 2.4. Post-Study Interviews

Two weeks after physical post-testing, short semi-structured interviews (7–10 min) were conducted to explore the varied compliance to the training. All participants were invited to participate in these exit interviews, irrespective of their attendance or completion of the program and were conducted at the participating school. Interviews were chosen in order to allow the participants the opportunity to authentically express their thoughts, feelings and perceptions of the program without ‘imposing’ prescribed answers and allowing for unexpected or novel responses to be explored [31]. To this end, an interview guide was developed (Supplementary File 1), based on Ryan et al.’s [32] interviews following a physiotherapy exercise intervention. Specifically, our key question underlying the current interviews was: What factors contributed to your engagement, adherence and enjoyment of the program? Participants reflected on their experiences during the intervention and reported what they liked and disliked. They were also invited to provide thoughts and suggestions for a future version of the training program.

### 2.5. Data Analysis

Descriptive statistics (means ± standard deviations) and individual results of all participants who completed pre- and post-data physical assessments were calculated. Because of the low number of athlete attendance at the post-test day, only descriptive measures across groups were deemed appropriate to report, rather than between-group statistical tests. A percentage change from each baseline test was calculated for individuals who attended the post-test in each of the physical capacity assessment and a whole point change for the MC tasks from the AIMS. To examine the individual responses we calculated the smallest worthwhile change (SWC) as 0.2 of the between-subject standard deviation from the whole cohort, as reported previously [21]. This SWC was expressed as a percentage of the mean to compare individual change. A frequency count established the number of individuals in each group who showed a change >SWC for each variable. Descriptive statistics were computed using SPSS Statistics (v.19, IBM SPSS Inc., Chicago, IL, USA), and the SWC and percentage change of individual scores calculated via an open-source Microsoft Excel spreadsheet [33].

Interviews were transcribed verbatim by the primary researcher (S.A.R.), with iterative, ongoing analysis using NVivo software (Version 12, QRS, Melbourne, Australia) beginning upon completion of the first transcription. Deductive content analysis was used to create codes and sub-categories, as per Elo and Kyngäs [34]. Trustworthiness (as described by Nayar and Stanley [35]) was confirmed at this point through independent verification of a random selection of interviews with A.H.R. Any disagreements in coding and/or placement of codes within sub-categories were resolved through discussion between S.A.R and A.H.R. Following agreement on code placement, sub-categories were placed into one of four pre-determined categories: motivation to join, engagement, post-study perceptions and suggestions for improvement (See Table 2 for sample coding).

## 3. Results

### 3.1. Physical Assessments

**Figure 2:**
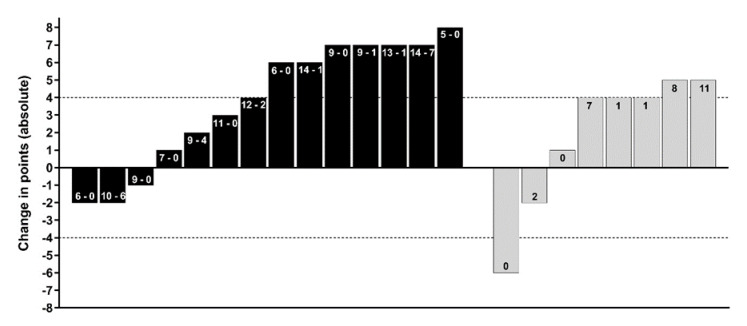
The absolute change in the Athlete Introductory Movement Screen (AIMS) sum score for the face-to-face group (black bars) and online prescription only (grey bars). Numbers within each black bar indicate training sessions completed from a possible 16 face-to-face sessions (first number) or 16 online sessions (second number). The number within grey bars indicate the number of self-directed sessions completed from a possible 32 prescribed over 16-weeks. Dashed horizontal lines indicate the smallest worthwhile change of 4 points (rounded to the nearest whole unit).

**Figure 3:**
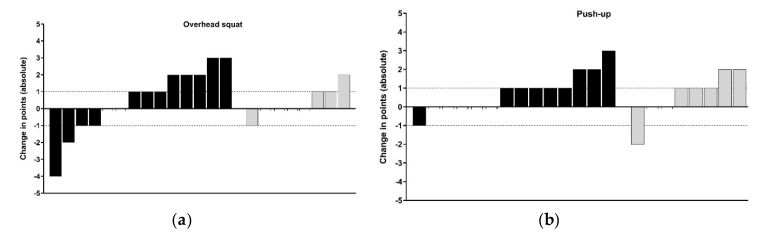

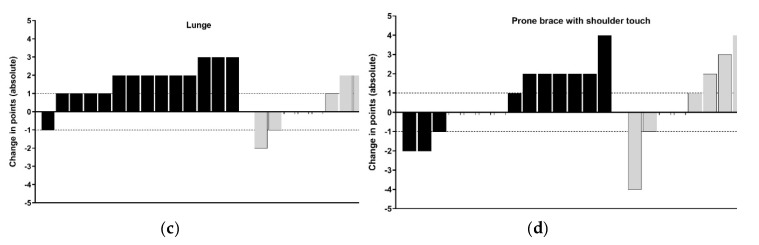
The absolute change in the Athlete Introductory Movement Screen (AIMS) individual assessment tasks for the face-to-face and online prescription (black bars) and online prescription only (grey bars) groups. (**a**) Overhead squat; (**b**) push-up; (**c**) lunge; (**d**) prone brace with shoulder touch. Dashed horizontal lines indicate the smallest worthwhile change of one point (rounded to the nearest whole unit).

**Figure 4:**
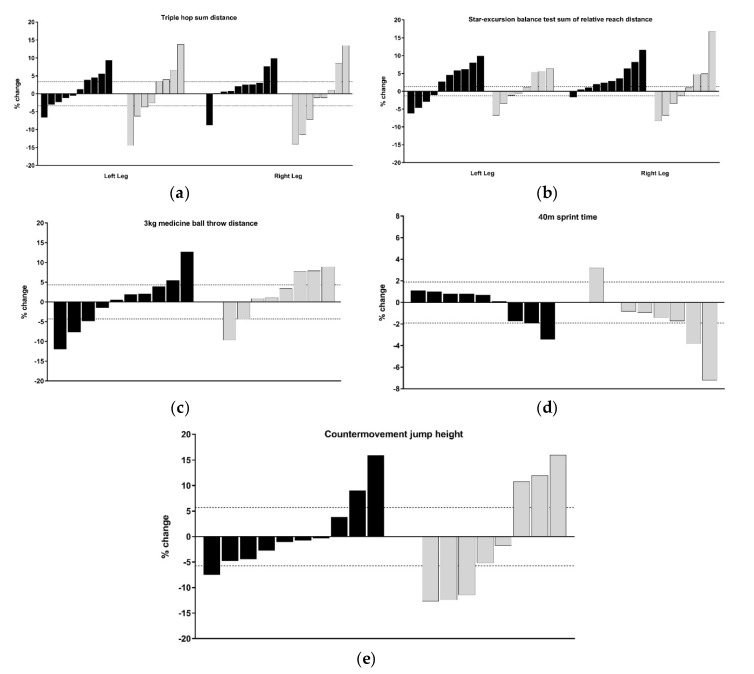
The percentage change for each physical capacity assessment for the face-to-face and online prescription (black bars) and online prescription only (grey bars) groups. (**a**) Triple hop sum distance; (**b**) star-excursion balance test’s sum of relative leg reach distance; (**c**) 3 kg medicine ball throw distance; (**d**) 40 m sprint time; (**e**) countermovement jump height. Dashed horizontal lines indicate the smallest worthwhile change as determined from pre-test data of all participants combined.

Group allocation, based on pair-matching participates from baseline MC (AIMS sum-score), led to a between group difference at baseline of only 0.9 points, which is < the SWC of on this primary outcome variable [22]. All participants who saw the study through and completed post-testing were included in the descriptive analysis, regardless of compliance. MC and physical capacity changes (whole points and percentage change) are presented in Figure 2, Figure 3 and Figure 4 using an individual responses approach. Figure 2 combines each athlete’s attendance rates for face-to-face sessions at school and compliance rates for self-directed sessions. Four athletes from the F2F group were absent from the post-test day, therefore, their AIMS post-testing was conducted within 7 days under the same conditions. However, because of scheduling conflicts, these four athletes could not complete the other physical capacity post-testing presented in Table 4. A summary of the raw scores of each respective movement task and physical capacity test are presented in Table 3 and Table 4 respectively. The number of participants per group that responded positively (*n* > SWC) in each physical capacity outcomes are also presented in Figure 3 and Figure 4. We deemed acceptable compliance as 75% as per previous work [15], with the addition of moderate compliance as 50–75%, with <50% deemed poor compliance.

Nine of the 22 participants who completed pre- and post-testing of the AIMS across the two groups, made improvements > SWC in the MC screen, with seven of these coming from the F2F group. As illustrated in the task-by-task breakdown of the raw change in AIMS tasks, the push-up showed the least improvement compared to the remaining three (Figure 3). In the physical capacity assessments (Figure 4), there were no observable differences between groups in the proportions of participants showing responses above or below the SWC. Of athletes who were allocated to and then attended the F2F training sessions, seven (50%) showed positive improvements > SWC in the sum-score of the AIMS. Attendance at F2F sessions varied from 31–88%, with a mean of 9.6 out of 16 possible lunch time sessions (60%). In the OL group, compliance with self-directed training was notably poor. Athletes from the OL group who completed pre- and post-physical testing (n = 8), logged between 0 and 11 sessions out of a possible 32 (mean of 12%).

### 3.2. Interview Findings

Interviews were performed to gain insights into four pre-identified areas of inquiry: motivation to join, engagement, post-study perceptions and suggestions for improvement. The findings provide context for the low compliance to training.

#### 3.2.1. Motivation to Join

A common theme found among participants was that many felt participating in the program was a viable opportunity for them to improve their physical capacities, and, in turn, their sporting performance.

‘I wanted to progress and get to high levels in my sport and I thought it would help me get there because it would build strength’. (Athlete 9, OL group).

Similar feelings were mirrored by participants who indicated they had signed up for the program in order to ‘get stronger and fitter quicker’ (Athlete 27, F2F group). Another key motivator was the novelty of the program and the chance to learn new skills. The athletes in this study were initially attracted by the chance to have a ‘new experience’ and to ‘see what’s going on’ (Athlete 23, OL group) with a sports research project within their school.

#### 3.2.2. Engagement

The compliant athletes indicated that internal factors, such as intrinsic motivation, were a significant part of their drive to complete the program:

‘I’m a bit of a try-hard, [laughs] I try to do well in everything, and I like sport… so yeah I wanted to do well in it’. (Athlete 20, F2F group)

Some also spoke about external support as being a large part of their continued engagement with the intervention. For example, one participant indicated that peer involvement was imperative:

‘Well, when I would do it with [participant A] and [participant B] and we would always text each other and be like ‘don’t forget we’ve got this on today’… but sometimes I would forget to check them, [email reminders] so it was good to have my friends saying that they would be coming to the training’. (Athlete 26, F2F group)

This positive social engagement came from both friends and family members, with family being a particularly strong source of motivation mentioned by some participants. Timing of sessions for the F2F group also had an influence on attendance and engagement, with at-school sessions engendering a positive response from the majority of participants:

‘I liked how it was in the middle of the day because you got to warm up it wasn’t straight in the morning, or it wasn’t at the very end when you’re tired, and you want to go home, so you’re kinda ready to do things’. (Athlete 20, F2F group)

Several barriers existed to realise an acceptable engagement level of 75% in the current cohort. Responses indicated that low engagement was predominantly due to a lack of time dedicated to completing the prescribed training. Participants made frequent mention of their already busy schedules when giving reasons for their low compliance with the self-directed sessions. Many in the F2F group noted the additional session requested to complete on their own each week (to compliment the at-school session), was the most difficult to accommodate, and had a lower priority than their sport-specific training. Athletes indicated that they struggled to ‘find time to do it [the second session online each week] with assignments and other sport’ (Athlete 25, F2F group).

Some participants openly acknowledged a lack of motivation and commitment to self-directed training, noting ‘I’ll admit… I slacked off a lot on it… I should have applied myself more’ (Athlete 17, OL group). Even the small subset of positive engagers in the school F2F sessions had limited compliance to the online sessions. Athlete 17 also noted, ‘sometimes I kind of put it off [second self-directed session] because of schoolwork and soccer training and stuff… so like I would only do it like once a week’. Several participants noted their engagement with online sessions was linked to a lack of training ‘enforcement’ from week to week.

#### 3.2.3. Post-Study Perceptions

The interviews also explored how the participants perceived the training program as a whole, both positive and negative. This included exploring the specifics of the exercises included in the training plans. In general, athletes felt the level of difficulty was satisfactory, with some athletes reporting they were extended in their abilities on some tasks.

‘Most of it was fine… nothing was too hard or too easy, most of it was technique stuff but that was what we were there for’. (Athlete 14, F2F group)

‘I thought the difficulty was really good because it was working on pretty basic things, but we really getting technical with each of them, so it was nice to slow things down and do things that we normally do’. (Athlete 12, F2F group)

Equally, several were willing to acknowledge that the difficulty experienced on some tasks was positive, providing them with physical challenges.

‘One thing I really liked was push-ups because I used to do them…. but when I was shown the proper way and where to put my arms it was really different and it was really good’. (Athlete 12, F2F group)

Some athletes felt the training assisted them from both a general physical confidence perspective, while also noting some perceived transfer between these physical improvements and their sport-specific domain:

‘…yeah, like gaining different skills and learning how to do the exercises and stuff properly… instead of just guessing’. (Athlete 25, F2F group)

‘I like the ones more to do with your legs because whenever I swim, my coach always tells me to use more of my legs, because I usually use my upper-body more, so the more leg movement activities I did, it felt like my legs were getting stronger in the water’. (Athlete 8, OL group)

There were, however, some participants who expressed a lack of challenge and believed the exercises were too simple, indicating they would have liked the exercises to be ‘a bit harder because it was easy to do most things’ (Athlete 4, F2F group). Another point raised by a handful of athletes was their initial perceptions of expecting more ‘fitness’-based sessions, rather than a focus on MC. Regarding the types of training performed, a small number of participants suggested sessions could be improved or the difficulty increased with more repetitions and volume.

‘I thought there would be a lot more fitness and like running and stuff in it, but there wasn’t too much of that so… but everything else was kind of what I expected’. (Athlete 22, F2F group)

#### 3.2.4. Improvement Suggestions

In order to gain deeper insights into the low training engagement, we prompted participants to provide their suggestions for a hypothetical future program. Responses were consistent around suggestions for alternative delivery methods of the program. When discussing the online access to the training plans, approximately a third of the participants offered for the prescription to be provided off-line or paper-based.

‘I reckon it would be better if you handed us a piece of paper for us to do, and then just fill in a diary, like instead of going online and looking through the exercises, but like get like a piece of paper’. (Athlete 23, OL group)

One participant noted how he found it frustrating not having a way of knowing if his technical competency in attempting an exercise was correct, without being able to draw upon in-the-moment feedback.

‘Some of the videos were hard to understand for some of the techniques, and without having someone there to correct you and yell at you if you’re doing something wrong, sometimes it’s hard and you start doing something wrong and it becomes like a habit and then you keep doing it cause it’s in the next exercise, and then next exercise… and you find out you’ve been doing it wrong the whole time.’

‘I’d rather do it at school because that way you’re with your friends, and they can like… give you constructive criticism and tell you what you’re doing wrong’. (Athlete 3, OL group).

The scheduling of our Monday lunchtime face-to-face sessions was a frequent topic of suggestions from the F2F interviewees. Suggestions included running multiple sessions each week or adjusting the time of the in-school sessions.

‘Maybe during like a little bit before school starts and then it running into home room [period] so it doesn’t interfere with class time, and people have assessments on [at lunch] and things like that’. (Athlete 12, F2F group)

‘Try probably give them [future participants] two days… like a Monday and a Wednesday, so if you miss one you’ve got the second chance to go again so you don’t miss out’. (Athlete 16, F2F group)

Several participants commented that they only wanted to perform all sessions at school; ‘I would have preferred two sessions at school… because it would have been easier’ (Athlete 5, F2F group). Conversely, a F2F group participant noted that, given a choice, they would have remained in the online group because ‘then I can do it at the gym with my family’ (Athlete 7, OL group). One participant also offered an insightful response and rationale for future group selection, highlighting the need for future interventions to carefully consider the cohorts’ for preferences when designing and delivering training:

‘I would ask them if they would rather do their training sessions at home alone, or see if they wanted to do them in a group at school… Cause then it gives them the choice, cos if they like doing it alone at home, they can do that, in their own time whenever they’re ready, but if you like doing it when you needed to do it at school with other people, then they might enjoy that one more’. (Athlete 8, OL group)

Finally, in order to improve their self-confessed low compliance to both forms of training, several participants proposed the inclusion of mobile phone reminder notifications to better support compliance with the self-directed sessions prescribed each week.

‘I think just like notifications that say like… you’ve been assigned new tasks you know… it includes this… do it by… like have deadlines sort of’. (Athlete 17, OL group).

## 4. Discussion

This study aimed to explore the effectiveness of and compliance with an introductory physical preparation program in a high-school setting. Incorporating both *process*- and *product*-orientated physical assessments, alongside qualitative findings from participant’s exit-interviews, we provide novel insights to improve future training prescription targeting MC for high-school athletes. Athletes who attended face-to-face coaching offered at school had greater compliance with sessions over self-directed sessions via an online platform. This F2F group had a larger proportion of participants demonstrate positive changes in MC.

Two recent studies [21,36] illustrated a range of individual responses in youth cohorts undertaking neuromuscular training interventions. Employing this reporting method in the present study, we report on the unique number of individuals from each group who were positive responders (i.e., a change > SWC) in the assessments used. Within the F2F group, half of the 14 athletes who completed a post-testing MC assessment, had improvements > 4 points (see Figure 2, black bars). These seven individuals came from a range of baseline competency levels, with their AIMS sum scores ranging from 27 to 38 points at pre-test. Notably, four of these positive responders also attended ≥9 of the 16 supervised training sessions offered. From the OL group, only two of eight who completed post-testing showed a positive change in AIMS score >4 points. These two individuals completed 8 and 11 of a possible 32 self-directed online sessions over the 16-week period (Figure 2, grey bars). This may suggest that without face-to-face coaching, high-school athletes may be less likely to engage with training prescriptions and thus plateau in a formal assessment of MC. Findings were supported by responses from the participant interviews related to the low compliance, as discussed in detail below.

The lunge task had the greatest number of participants exhibiting positive changes (64% of the F2F group), while only three F2F and two OL participants improved in the push-up by more than 1 point (see Figure 3b,c). Thus, when low levels of training were completed, the current cohort of high-school athletes’ MC (both sum and individual task scores of the AIMS) remained within the normal scoring error reported for this tool [22]. Conversely, athletes who engaged in moderate levels of F2F sessions (see Figure 2, black bars), improved MC scores across most of the four athletic preparation tasks in 16 weeks. It is well accepted that responses to training are influenced by the intensity, frequency, and volume of training in youth [18]. Across the current intervention, the percent change from five commonly employed physical capacity assessments (detailed in Section 2.2.3, Section 2.2.4, Section 2.2.5, Section 2.2.6 and Section 2.2.7) were inconclusive, as the majority of individuals change scores sat within the SWC (Figure 4). This could be explained as a result of the intervention focus being on the acquisition and practising of correct movement techniques, rather than amassing the higher training volumes and intensity levels, the latter of which may be required to elicit improvements across muscular fitness qualities. As the present intervention had an experimental group who only had access to video demonstrations of prescribed exercises (i.e., self-coached), sessions were set at safe levels of difficulty based on observed low levels of competency at baseline. This follows the recommendations of Myer, Faigenbaum, Ford, Best, Bergeron and Hewett [3] who noted the initial volume should be low, allowing an athlete to learn the skill to perform each task with proper technique.

A key finding of this study was that compliance with online training sessions was generally low, regardless of the intervention group (Figure 2). Interviews revealed that compliance was closely linked to enjoyment and novelty of the program. In another study of high-school students, Lubans, Sheaman and Callister [14] investigated training modalities to improve muscular fitness and body composition (resistance bands compared to free weight training and a control group). Across their two intervention groups, the authors reported 73–79% attendance rates and cited additional academic and extra-curricular commitments as potential reasons for failing to attend lunchtime sessions. However, in the present study, we expanded on the hypothesised reasons offered by Lubans et al. [14], and collated, via interviews, participants’ reasons for their overall lack of adherence.

In our study, it was not possible to set-up the F2F group with training scheduled during a dedicated class period. While all sessions were noted in the information to participants and parents as voluntary, the coordinating teacher and lead researcher provided regular session reminders and encouragement via both student and parent email. From the interviews, we found a number of comments relating to the low or non-compliance of participants. In particular, their perceived lack of time to complete the sessions each week, was common regardless of the delivery method allocation. These comments regarding time pressures were supported by relatively high hours of the participants self-reported sporting commitments, which were recorded at the commencement of the study (Table 1). Potentially aligned with the lack of time to dedicate to physical preparation training, were participants’ perceptions following the study. These included lacking motivation and/or enjoyment during self-directed sessions over the school sessions. Ultimately, it appeared that the majority of students who attempted to attend the at-school training when possible, did enjoy the face-to-face sessions on offer. When asked, many athletes stated that they would like more of coach-led sessions in future programs. A noteworthy finding from this pilot study was that some participants reported positive perceptions to the OL self-directed delivery, such as flexibility to fit in at a suitable time, and the ability to incorporate the training into family gym sessions.

The importance of ‘buy-in’ of physical preparation programs was discussed within the Positive Youth Development framework [37]. Athlete experiences are enhanced when they trust that the methods used by a coach will support them to achieve their sporting ambitions. Our recommendations to improve adherence, align with those suggested by Lubans et al. [14]. That is, future studies should include goal setting principles, innovative session reminder strategies such as mobile phone notifications, enhanced social support (e.g., structure and deliberate parental involvement), and targeted education around how physical preparation transfers to sport. It is possible that only some participants felt performing the prescribed training was transferable to supporting their sporting performance, as few mentioned of this in exit-interviews. We propose, therefore, that including the athlete’s sport-specific coach into the delivery of physical preparation (rather than external providers) may enhance buy-in and, ultimately, athlete adherence. Sports coaches may be the most influential contributor to youth sporting environments and have been acknowledged as one of the most influential adults in a child’s life after their parents [38]. Accordingly, delivery methods for youth physical training should continue to be explored regarding how MC skills can be packaged and delivered by more youth sport coaches. By providing these coaches with best-practice MC session templates and appropriate progressions, athlete exposure to adequate physical preparation can be maximised. Notably, coaches who hold a positive attitude towards injury prevention, have been reported to deliver an injury prevention strategy (inclusive of improving movement competency and neuromuscular capacity), and this may be a crucial aspect to athlete compliance and ultimately the success of a programme in youth sports [39]. The context of the present lunchtime training program (led by a researcher external to the athlete’s sports coach or team), could be a factor underlying the low compliance, wherein the athletes were from a mixture of sports, and the program was not lead nor requested by their sports coach.

Learning a new skill involves athletes exploring a range of strategies for achieving the required body positions and movement sequences of a task. Within skill acquisition literature, this is known as the cognitive or coordination stage [40]. As a novice athlete puts together a mental model of a task (e.g., sequencing of the hip hinge in a squat), and while in the early phases of learning, they are required to pay a significant level of attention to this deliberate and step-by-step process. There are typically many movement errors at this initial stage, and importantly the novice and/or younger athlete [41] may require substantial feedback from demonstrations, along with appropriate and short cue words to progress to their next stage of improved movement. Across our post-study interviews, several participants mentioned their desire for this form of feedback as part of the training process. In the current study, demonstrations of the physical preparation and MC skills were provided as two-dimensional videos with text-based cues for online training. Videos did not have a coach voice-over or auditory cue for athletes to use; thus an absence of concurrent cues being delivered during the learning phase, in addition to a lack of coach feedback, could be a disadvantage of this delivery method. This may offer a further explanation for the lack of positive improvement in MC for OL participants. While the videos in the present study did have some positive feedback comments from participants, the design and teaching principals of video-based coaching is an area worthy of further consideration.

While a large body of literature has discussed physical capacity and performance improvements following neuromuscular training in youth athletes, seldom is there inclusion of developmentally appropriate measures of MC, or reporting on the technical assessment of the skill required to perform movements safely. We speculate that the acquisition of movement techniques may be effectively influenced by relatively brief training exposure and low-intensity training. From several of the individual responses to the AIMS in the current study, some individuals who only attended a small number of sessions were nevertheless, able to make meaningful improvements in selected MCs. This study should encourage future researchers to include a ‘learning phase’ for MCs required as part of a physical preparation strategy. This could include a measurement tool such as the AIMS to assess MC during a lead-in or baseline period prior to more complex training stimuli (i.e., external loading). Such a lead period could be performed in conjunction with familiarisation of other performance tests (often maximal) and allow for a more robust understanding of changes that occur as a result of an intervention. Future studies may also look to explore ways to improve adherence to non-sport specific physical preparation programs. For example, exploring the influence of delivery personnel on MC outcomes (e.g., sport coach, strength and conditioning coach or parent followings instructional training to supported remote athletes). Finally, future work in this area may look to incorporate the use of novel tools to track compliance and provide regular updates to the athlete’s themselves or their stakeholders such as parents, teachers or coaches.

The following limitations, however, need to be considered within the outcomes of this pilot study. First, while groups were initially formed based on pair-matching of MC scores at baseline, the mean baseline scores of the two groups who continued to study to completion (Table 3), were greater than the initial between group baseline differences that were reported in Section 3.1. Therefore, future studies require greater emphasis on participant continuity from pre- to post-testing when researchers are not embedded within the high-school cohort. Second, while we attempted to standardise the workloads of each training session with the 16-week program, there were periods where, due to maturation and gender differences, some athletes were able to progress their learning and practicing of MC tasks with external load (dumbbells and light barbells) sooner than others. Furthermore, the current study took place over the competitive season for the participants from mixture of sports (e.g., Australian football, field hockey, netball, soccer, swimming), and thus the differences in sports-specific training stimuli outside of the study prescription varied. Future work could attempt to minimise this limitation by focusing within a single sport cohort who train under the same sport-specific routine and personal. While we collated the self-reported sports participation data in line with other established research protocols (see [42,43]) we acknowledge the limitation of participants potential over- or under-estimation of exposure hours via self-report. Finally, while this pilot work has added novel insights from high school athlete perceptions via our semi-structured interview analysis, one area to improve these insights in future work could be to corroborate the adherence challenges noted by athletes with parent/guardian feedback. With participants instructed to complete sessions at home, parents/guardians may have offered insightful first-hand observations from observing their children attempt to improve MC and physical capacities.

## 5. Conclusions

Through this pilot study, we have presented novel insights that explore how high-school athletes can improve MCs with appropriate training. Improvement in MC was seen in a 16-week program for those who attended face-to-face training. Compliance across both F2F and OL groups appeared aligned to MC changes, with face-to-face delivery offering clear advantages in terms of both increased compliance and improvements in movement skill. This may be linked to F2F athletes receiving technical feedback during key learning phases of movement. Regardless of the delivery group, physical capacities outside of MC scores were not affected with the current program, likely because of low training intensities, owing to the technical focus of the sessions and overall poor compliance. Thus, the self-directed training intervention approach has limitations related to athlete motivation and the lack of feedback during the learning process. Findings from the current qualitative responses, allow researchers and practitioners to consider perceived barriers to training programs in future work with high-school athletes. Youth sports practitioners and sporting organisations, therefore, may benefit from prioritising face-to-face coaching delivery during the early phases of acquiring competency in strength training techniques, while continuing to record and reflect on the thoughts of the participants after such physical preparation interventions.

## Figures and Tables

**Table 1:** Participant-reported hours in physical activity of all participants at intervention commencement.

Baseline Group Allocation	Free Play/Week (hours) Mean ± SD (Range)	Planned Training/Week (hours) Mean ± SD (Range)	Planned Competition/Week (hours) Mean ± SD (Range)
F2F (*n* = 18)	5.3 ± 5.5 (0.0–20.0)	6.5 ± 3.8 (1.0–14.0)	2.6 ± 1.3 (0.7–5.1)
OL (*n* = 21)	5.0 ± 4.6 (0.0–17.1)	7.9 ± 5.2 (2.0–19.5)	4.0 ± 3.4 (1.0–13.0)

**Table 2:** Interview coding with meaning unit examples.

Example Meaning Unit	Example Code	Example Sub-Category	Category
‘I wanted to, like, improve my fitness and get better at training so I’d get better at doing my sport’.	Improve fitness (general)	Physical Improvement	Motivation to join
‘Sometimes I kind of put it off because of schoolwork and soccer training and stuff so I would only do it like once a week’.	Conflicting priorities	Lack of time	Engagement (or lack of)
‘Yeah definitely like some of the stuff about deadlifting and stuff, so I wasn’t very good at the technique or anything, and um yeah it was helping me when joining a gym’.	Technical/skill improvement	Perceived benefits	Post-study perceptions
‘Umm I reckon it would be better if you handed us a piece of paper for us to do, and then just fill in a diary, like instead of going online and looking through the exercises, but like get like a piece of paper’.	Paper copy of program	Offline programming options	Improvement suggestions

**Table 3:** Individual task and sum-scores from the Athlete Introductory Movement Screen (AIMS) for participants completing pre- and post-assessment.

Group	Group 1: F2F (n = 14)			Group 2: OL (n = 8)		
Task	PRE Mean ± *SD*	POST Mean ± SD	% > SWC	PRE Mean ± SD	POST Mean ± SD	% > SWC
**Overhead squat**	7.9 ± 1.9	8.4 ± 2.1	36%	7.6 ± 1.6	8.0 ± 1.6	13%
**Push-up**	9.9 ± 1.3	10.7 ± 1.0	21%	8.6 ± 2.2	9.3 ± 2.6	25%
**Lunge**	8.0 ± 1.7	9.4 ± 1.5	64%	8.5 ± 1.8	8.8 ± 2.1	25%
**Brace with shoulder touch**	8.7 ± 1.8	9.4 ± 1.5	43%	8.1 ± 1.8	8.8 ± 1.5	38%
**AIMS sum score**	34.5 ± 4.2	38.3 ± 4.8	50%	32.9 ± 4.7	34.8 ± 3.3	25%

Each of the four tasks above is scored out of a possible 12 points, and AIM sum-score out of 48 (see [22]). F2F = Intervention with one session/week face-to-face; OL = Intervention with online sessions only; *%* > SWC = relative number of participants with the group who showed a positive change greater than the smallest worthwhile change (SWC) of 1 point on for each movement OR 4 points for the sum score.

**Table 4:** Change scores of physical capacity measures.

Group (*n* Completed Pre and Post)	SWC (% of the Pre-Test Mean)	Group 1: F2F (n = 10)				Group 2: OL (n = 8)			
		PRE Mean ± SD	POST Mean ± SD	Δ Mean % ± SD	n > SWC	PRE Mean ± SD	POST Mean ± SD	Δ Mean % ± SD	**n > SWC**
Triple hop total left leg (m)	0.17 (3.5%)	5.05 ± 0.86	5.11 ± 0.93	1.1 ± 4.7	4	4.51 ± 0.78	4.53 ± 0.93	0.1 ± 8.7	4
Triple hop total right leg (m)	0.16 (3.2%)	5.04 ± 0.73	5.16 ± 0.84	2.1 ± 4.9	2	4.68 ± 0.64	4.63 ± 0.85	−1.4 ± 9.4	2
3 kg medicine ball throw (m)	0.17 (4.3%)	4.11 ± 1.11	4.07 ± 0.95	0.1 ± 7.0	2	3.71 ± 0.80	3.79 ± 0.83	2.0 ± 6.5	3
SEBT left leg composite (cm)	1.1 (1.3%)	84.8 ± 5.7	86.6 ± 4.5	2.2 ± 5.3	6	85.3 ± 4.1	85.9 ± 4.2	0.8 ± 4.7	3
SEBT right leg composite (cm)	1.3 (1.5%)	85.2 ± 4.9	88.3 ± 5.7	3.7 ± 3.8	7	84.9 ± 4.9	85.5 ± 5.0	1.0 ± 8.0	3
CMJ height (cm)	1.6 (5.7%)	27.0 ± 6.0	26.9 ± 5.0	0.8 ± 7.1	2	23.2 ± 6.0	22.8 ± 5.6	−0.5 ± 11.9	3
40 m sprint time (s)	0.12 s (1.9%)	5.96 ± 0.56	5.94 ± 0.50	−0.3 ± 1.6	1	6.57 ± 0.53	6.47 ± 0.58	−1.6 ± 3.0	2

F2F = Intervention with one session/week face-to-face; OL = Intervention with online sessions only; n > SWC = number of participants from the group who showed a change greater than the smallest worthwhile change calculated from all pre-test scores; SEBT = star excursion balance test; CMJ = countermovement jump.

## Supplementary Materials

The following are available online at https://www.mdpi.com/2075-4663/8/4/39/s1, caption: Semi-structured interview questions and prompts.

## Appendix A

Boys: peak height velocity predictive equation as per [26]:

Maturity Offset = −9.236 + 0.0002708·Leg length and sitting height interaction −0.001663·Age and Leg Length interaction + 0.007216·Age and Sitting Height interaction + 0.02292·Weight by Height ratio (A1).

Girls peak height velocity predictive equation as per [26]:

Maturity Offset = −9.376 + 0.0001882·Leg Length and Sitting Height interaction + 0.0022·Age and Leg Length interaction + 0.005841·Age and Sitting Height interaction − 0.002658·Age and Weight interaction + 0.07693·Weight by Height ratio (A2).

## Appendix B

**Table A1:** Example training sessions set for both groups.

Session 1	Exercise	Sets	Reps	Session 2	Exercise	Sets	Reps
Plyometric intro + land technique	Lateral jumps across a line	3	5/side	Locomotor skills	High knee walking ‘A’ march	3	15 m forward
	Hop, ¼ turn and stick	3	5/side		Skipping ‘A’ drill	3	15 m forward
MC lower body	Squat—overhead dowel rod	3	5	Landing + force control	Countermovement jumps with soft landing	3	5
	Deadlift—barbell	3	5	Bracing	Supine plank	3	30 s
	Lunge—travelling	3	10/side		Side plank	3	30 s
MC upper body	Kneeling push-ups	3	5		Prone plank with hands on bench	3	30 s
