# Supplementary material for: Movement Competency Training Delivery: At School or Online? A Pilot Study of High-School Athletes
Source: Sports (Basel). 2020 Mar 26;8(4):39. doi: 10.3390/sports8040039 (PMC7240720; doi:10.3390/sports8040039)
Supplement: Supplementary file 1 [file sports-08-00039-s001.pdf]

sports

Supplementary Materials

Movement Competency Training Delivery: At School or Online? A Pilot Study of High-School Athletes

Simon A Rogers [1,2,*], Peter Hassmén [1], Alexandra H Roberts [2,3], Alison Alcock [1] and Wendy L Gilleard [1] and John S Warmenhoven [2,4]

S1: Semi-structured Interview Questions and Prompts

Adapted from Ryan et al. (2016)

1. Overall participation

In your own words what was it like being part of the project?

Prompts:

- Can you tell me what you liked?
- What were your original reasons for taking part?
- What you disliked?
- What was easy or hard?
- What were the challenges?
- How did you find the length of time required by the programme?
- Would you have preferred longer/shorter sessions? More or less?

2. At school training sessions

In your own words can you tell me about your experience of taking part in the face-to-face training sessions as part of this study?

Prompts:

- How did the class/session compare to anything you've done before?
- How did you find the specific exercises?
- Were the exercises too easy or too difficult for you?
- Anything you particularly disliked about it? - Why?
- Did you have any problems/adverse effects while doing you training either in the class?
- Do you now feel more comfortable using gym equipment after these sessions? Why so/why not?

3. At home training sessions

In your own words can you tell me about your experience of taking part in the class as part of this study?

How did the home training programme compare to anything you've done before?

Prompts:

- How did you find the specific exercises?
- Is there anything you would change?
- Were the exercises too easy or too difficult for you?
- Anything you particularly disliked about it? Which activities did you like the least?
- Did you have any technical problems viewing the demos while doing you training at home?
- How did you find completing the exercises on 2 days a week?
- Did you feel comfortable completing the program at home
- Do you now feel more comfortable using gym equipment after these sessions? Why so/why not?

4. Using the training platform (website)

What was it like using the website as part of your training?

Prompts:

- Have you ever used any form of technology to help you with exercise or training before?
- Do you think the website made a difference to how often and how long you trained for? In what way?

5. Previous Training Experience vs. current project

Before taking part in this project, what sort of exercises did you do?

Prompts:

- How often, amount of time, home or gym exercise programme, type of exercises.

6. Other

- Any other comments about any aspect of the study?
- What would be your main piece of advice for someone running a training programme like this?
- In your opinion, what have been the main benefits and disadvantages of taking part in this study?

7. General Prompts and Probes

- How did that make you feel?
- That's interesting can you tell me more about that?
- Can you elaborate a little more?
- Could you clarify that?
- I am not quite sure I understand. You were saying?
- When you say …………….. Did you mean that………..
